# N-octanoyl-Dopamine Is an Agonist at the Capsaicin Receptor TRPV1 and Mitigates Is Chemia-Induced Acute Kidney Injury in Rat

**DOI:** 10.1371/journal.pone.0043525

**Published:** 2012-08-20

**Authors:** Charalambos Tsagogiorgas, Johannes Wedel, Maximilia Hottenrott, Michael O. Schneider, Uta Binzen, Wolfgang Greffrath, Rolf-Detlef Treede, Bastian Theisinger, Sonja Theisinger, Rüdiger Waldherr, Bernhard K. Krämer, Manfred Thiel, Peter Schnuelle, Benito A. Yard, Simone Hoeger

**Affiliations:** 1 Department of Anaesthesiology and Intensive Care Medicine, University Medical Centre Mannheim, Medical Faculty Mannheim, Ruprecht Karls University Heidelberg, Mannheim, Germany; 2 Department of Medicine V (Nephrology/Endocrinology/Rheumatology), University Medical Centre Mannheim, Medical Faculty Mannheim, Ruprecht Karls University Heidelberg, Mannheim, Germany; 3 Division of Neurophysiology, Center of Biomedicine and Medical Technology Mannheim (CBTM), Medical Faculty Mannheim, Ruprecht Karls University Heidelberg, Mannheim, Germany; 4 Novaliq GmbH, Heidelberg, Germany; University of Sao Paulo Medical School, Brazil

## Abstract

Since stimulation of transient receptor potential channels of the vanilloid receptor subtype 1 (TRPV1) mitigates acute kidney injury (AKI) and endogenous N-acyl dopamine derivatives are able to activate TRPV1, we tested if synthetic N-octanoyl-dopamine (NOD) activates TRPV1 and if it improves AKI. These properties of NOD and its intrinsic anti-inflammatory character were compared with those of dopamine (DA). TRPV1 activation and anti-inflammatory properties of NOD and DA were tested using primary cell cultures *in vitro*. The influence of NOD and DA on AKI was tested in a prospective, randomized, controlled animal study with 42 inbred male Lewis rats (LEW, RT1), treated intravenously with equimolar concentrations of DA or NOD one hour before the onset of warm ischemia and immediately before clamp release. NOD, but not DA, activates TRPV1 channels in isolated dorsal root ganglion neurons (DRG) that innervate several tissues including kidney. In TNFα stimulated proximal tubular epithelial cells, inhibition of NFκB and subsequent inhibition of VCAM1 expression by NOD was significantly stronger than by DA. NOD improved renal function compared to DA and saline controls. Histology revealed protective effects of NOD on tubular epithelium at day 5 and a reduced number of monocytes in renal tissue of DA and NOD treated rats. Our data demonstrate that NOD but not DA activates TRPV1 and that NOD has superior anti-inflammatory properties in vitro. Although NOD mitigates deterioration in renal function after AKI, further studies are required to assess to what extend this is causally related to TRPV1 activation and/or desensitization.

## Introduction

Acute kidney injury (AKI) is common in critically ill patients and is associated with a substantially increased morbidity and mortality [1], [2]. Although the etiology of AKI is often multifactorial, major causes include renal hypoperfusion, direct nephrotoxicity, systemic inflammatory response syndrome (SIRS) and sepsis [3], [4]. Inflammation is believed to play a major role in the pathophysiology of AKI [5], [6]. Inflammatory processes lead to endothelial disintegration with an increase of vascular permeability, overexpression of adhesion molecules accompanied by infiltration of neutrophiles and macrophages into the renal parenchyma. In AKI, loss of renal function is attributed to severe tubular injury as a consequence of an increased amount of reactive oxygen radicals, leading to apoptosis and necrosis [7], [8]. In addition to their passive role in AKI, proximal tubular epithelial cells also produce numerous chemokines upon oxidative stress, which further perpetuates ongoing inflammation [9].

For many decades low-dose dopamine (DA) has been applied for prevention and treatment of AKI in critically ill patients [10], [11]. However, several meta-analyses and prospective studies have concluded that DA treatment neither prevents nor ameliorates AKI in these patients [12], [13], [14], [15], [16]. This is in contrast to renal transplant recipients, where the use of low- dose DA in the donor reduces the incidence of delayed graft function and consequently improves long-term graft survival in the recipient [17]. Donor DA treatment attenuates the increased immunogenicity of organs following brain death and leads to a reduction of renal inflammation as has been shown in *in vitro* and *in vivo* models [18], [19], [20].

**Figure 1:**
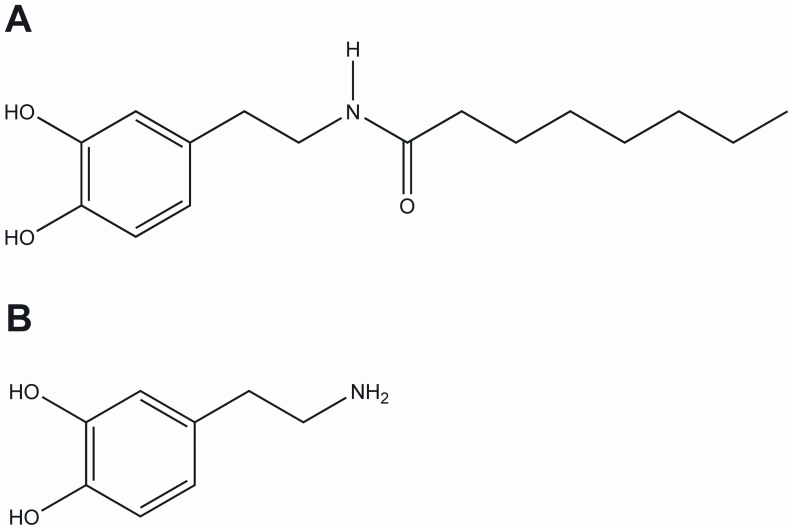
Chemical structures. **A. Dopamine derivate N-octanoyl-dopamine (NOD)** Modification of dopamine was performed by coupling octanoic acid at the amine side chain of dopamine **B. Dopamine.**

N-acyl dopamine derivatives (NADD) have recently come to attention as endogenous compounds with biological activities in the nervous system, vasculature and the immune system [21]. They are described as effective activators of transient receptor potential channels of the vanilloid receptor subtype 1 (TRPV1) [21], [22]. Like other TRPV1 agonists, they have the potential to prevent and attenuate ischemia/reperfusion (I/R) injury in different organs including kidney [23], [24]. A number of TRPV1 agonists can also mediate immune modulation independent of TRPV1, via a mechanism involving NFκB [25], [26], [27], [28], [29], [30].

**Figure 2:**
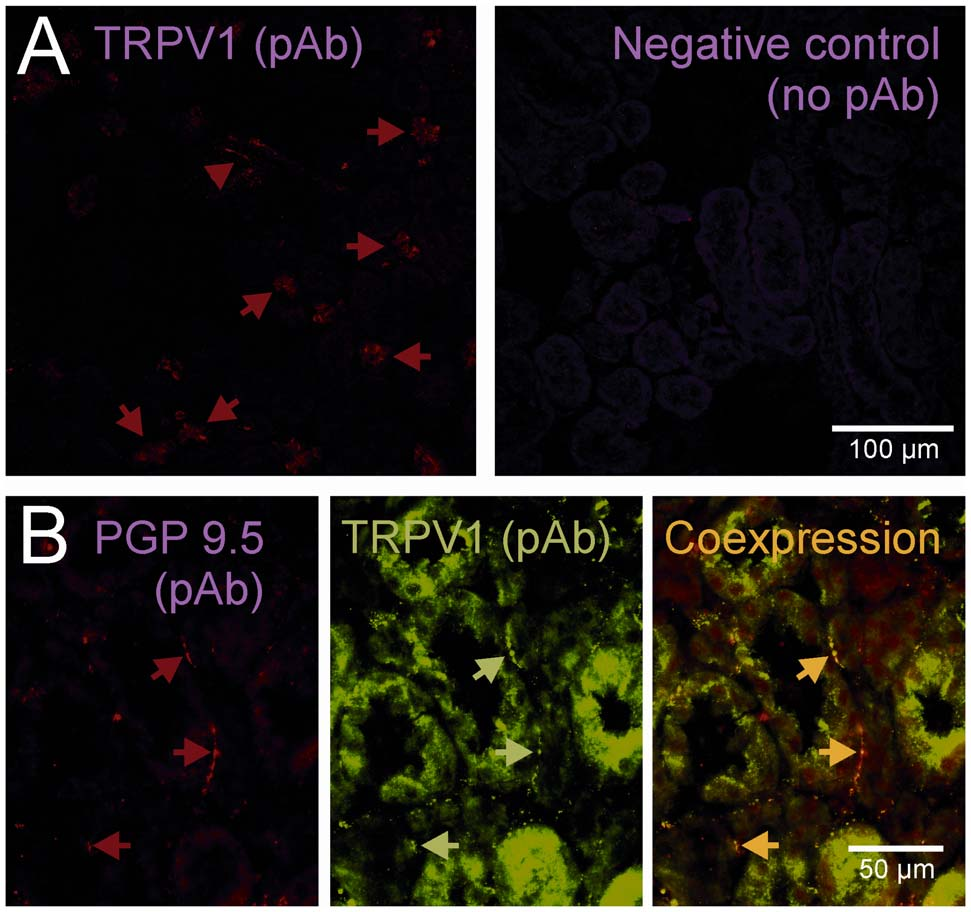
Expression of TRPV1 in rat renal tissue. **A.** Cryostat sections of rat renal tissue were stained for TRPV1 by indirect immunofluorescence using polyclonal an antibody (left). Note that distinct tubular structures were positive for TRPV1 (arrows). Omission of the primary was used as negative control (right). **B.** At higher magnifications it was observed that PGP9.5 positive nerve fibers (arrows) within the rat kidney also express the capsaicin receptor TRPV1 as shown by the overlay of both.

The synthetic N-octanoyl dopamine (NOD) [31] belongs to the class of NADD and has structural similarities to the TRPV1 agonist capsaicin. Compared to DA, NOD shows enhanced cellular uptake and is on a molar basis approximately 40-fold more effective in protecting endothelial cells against cold preservation injury [31]. It is however currently not known if the synthetic NOD can activate TRPV1, similar as has been described for the endogenous members N-arachidonoyl-dopamine and N-oleoyl-dopamine. Likewise, its anti-inflammatory potential and whether or not it can mitigate AKI have not been addressed thus far. In the present study we tested the hypothesis that unlike DA, NOD activates TRPV1 and has superior anti-inflammatory properties *in vitro*. Hence *in vivo*, NOD might improve renal function in a setting of ischemia induced AKI.

**Figure 3:**
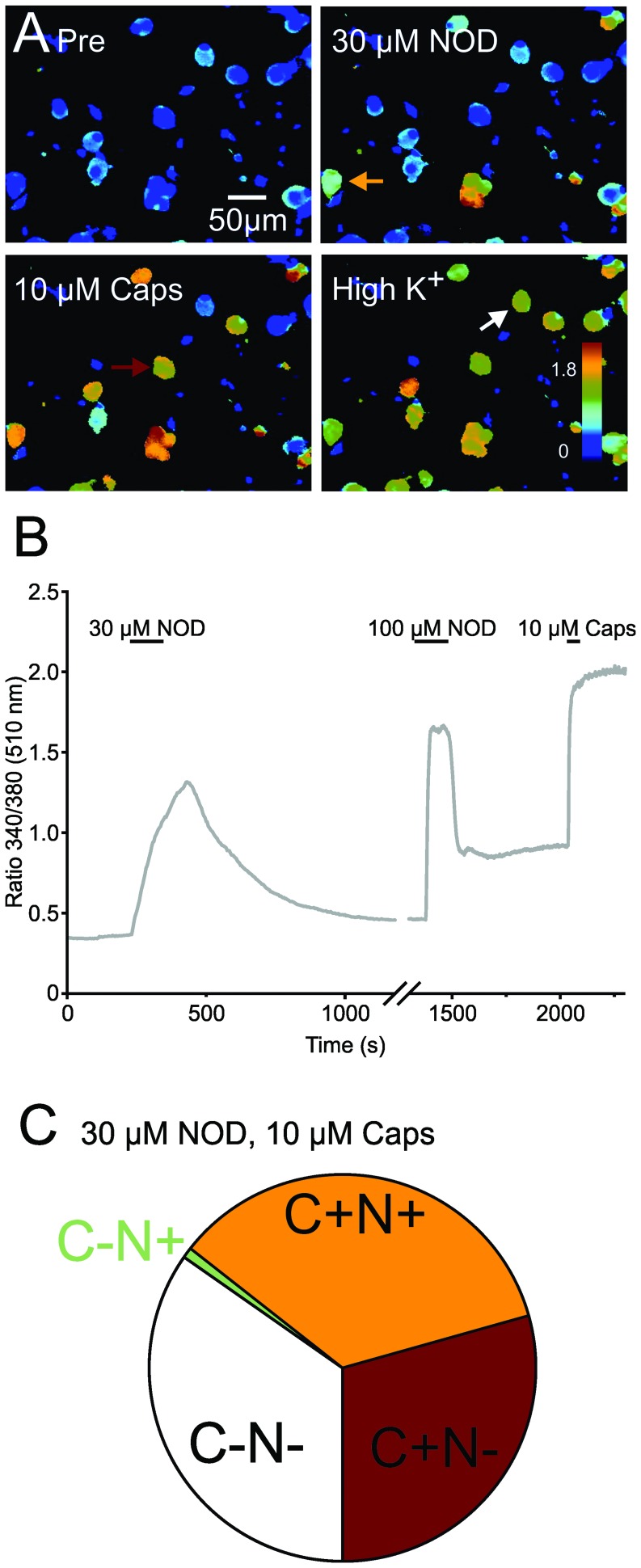
NOD activates capsaicin-sensitive rat dorsal root ganglion neurons. **A.** Representative example of a calcium imaging experiment using rat DRG neurons (see upper left). The ratio 340/380 at 510 nm – a measure proportional to free intracellular calcium [33] – is false colour coded where warmer colours indicate increasing calcium values. Representative cell responses are shown in **B.** to application of 30 µM NOD, 100 µM NOD and 10 µM capsaicin. **C.** Frequencies of DRG neurons (n = 499 obtained in 9 experiments) responding to 30 µM NOD and to capsaicin (10 µM, yellow), to capsaicin only (red) and neither to capsaicin nor NOD (open).

## Methods

### Chemicals

All chemical reagents were purchased from Sigma Aldrich (Sigma-Aldrich Chemie GmbH, Munich, Germany) unless otherwise indicated.

**Figure 4:**
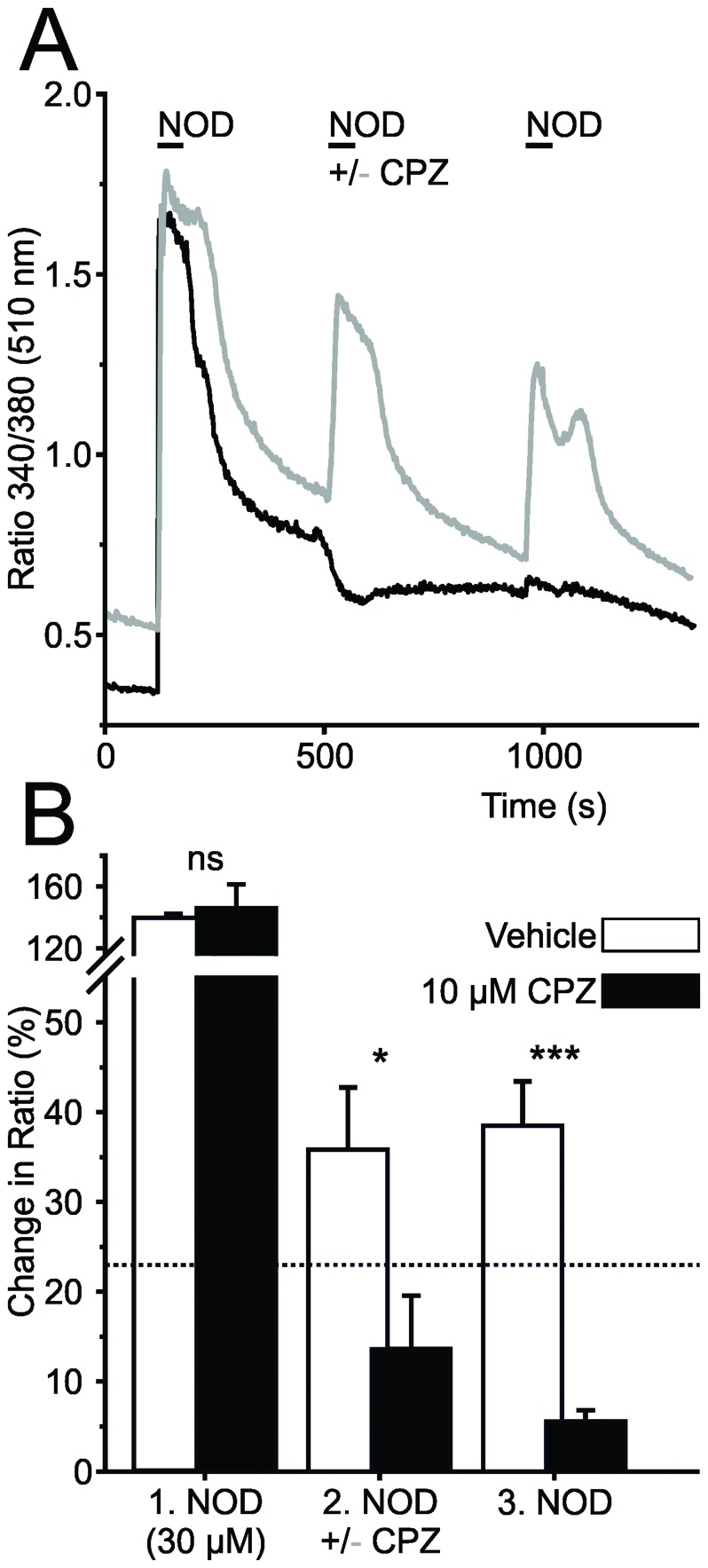
NOD activates TRPV1 in DRG neurons. **A.** Increases in free intracellular calcium in response to repetitive application of NOD displayed marked tachyphylaxis from stimulus to stimulus (grey trace). Application of the competitive TRPV1 antagonist capsazepine before and during the 2^nd^ stimulus (CPZ; black trace) abolished the NOD response. **B.** Mean change in intracellular calcium by NOD in cells challenged with (filled bars; n = 4) and without CPZ during the second stimulus (open bars; n = 5) indicated the specificity of NOD at TRPV1. The threshold for a significant response is indicated by the dotted line (*p<0.05, ***p<0.001 vehicle versus CPZ, student’s unpaired t-test).

### Animals

Inbred male Lewis (LEW, RT1) rats weighing 200 to 250 g were obtained from Charles River (Sulzfeld, Germany), acutely dissociated DRG neurons and tissue for TRPV1 immunostaining was obtained from male Sprague-Dawley rats (Janvier, France). Animals were kept under standard conditions and fed standard rodent chow and water ad libitum. All procedures were performed according to the Guide for the Care and Use of Laboratory Animals published by the National Academy of Sciences and were approved by the local authorities (Regional council Karlsruhe, Germany; reference number 35–9185.81/G 61/05).

**Figure 5:**
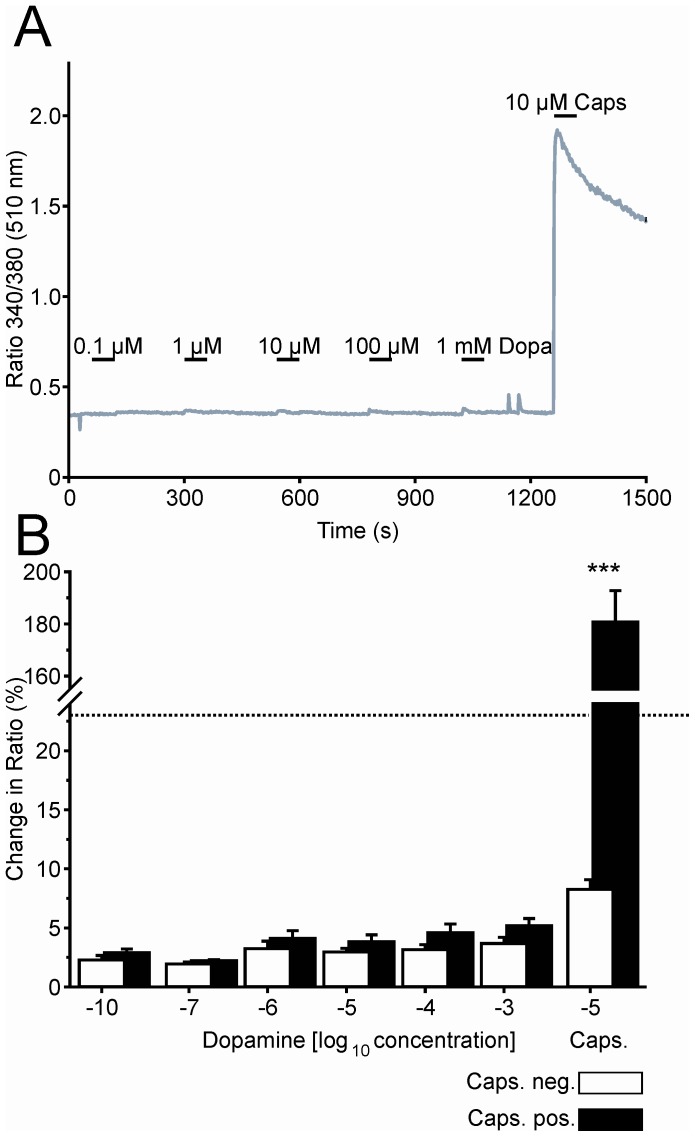
Dopamine does not activate capsaicin-sensitive DRG neurons. **A.** Representative examples of calcium imaging experiment using a capsaicin-sensitive DRG neuron challenged with increasing doses of dopamine (0.1–1000 µM). B. Neurons were separated in capsaicin-sensitive (black) and capsaicin-insensitive neurons (open bars). The threshold for a significant response is indicated by the dotted line. 595 excitable neurons investigated in 8 independent experiments (***p<0.001 Capsaicin-sensitive versus –insensitive neurons, student’s unpaired t-test).

### N-octanoyl Dopamine (NOD) Synthesis

NOD (Fig. 1 A) was synthesized from commercially available precursors and was purified by twofold recrystallization from dichloromethane as demonstrated by thin layer chromatography (TLC). Octanoic acid was converted to its mixed anhydride derivate by reaction with ethyl chloroformate in the presence of N-ethyl diisopropylamine. The crude mixed anhydride was incubated with dopamine hydrochloride in N,N-dimethylformamide and N-ethyl diisopropylamine to form NOD. After aqueous preparation and evaporation of the organic solvent NOD in an overall yield of approximately 60% is obtained. The sample investigated by NMR (Bruker AC250) yielded spectra in accordance with the expected structure.

**Figure 6:**
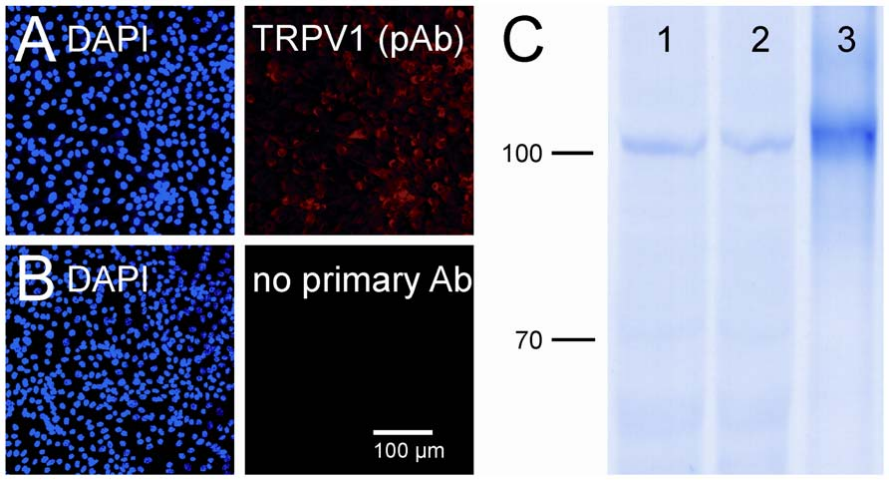
TRPV1 expression in human cultured PTEC. **A.** Human PTECs were marked with DAPI and stained with a primary anti-TRPV1 antibody (giunea-pig polyclonal serum); omission of a primary antibody served as negative control (**B**). **C.** In addition TRPV1 was assessed in lysates of unstimulated (1) or TNF-α stimulated PTECs (2) by Western blotting. TRPV1 transfected HEK cells (3) were used as positive control to indicate the molecular weight of TRPV1.

### Activation of Primary Nociceptive Neurons by NOD

Preparation of acutely dissociated DRG neurons and intracellular calcium measurements were done similar to methods described before [32] with a few alterations: Dorsal root ganglia (DRG) were dissociated enzymatically and mechanically using collagenase (5 mg/ml; CLS II; Biochrom, Berlin, Germany) and accutase (PAA, Coelbe, Germany). Neurons were resuspended in neurobasal medium (Invitrogen, Darmstadt, Germany) containing 2% horse serum, B27 (Invitrogen), 100 IU ml-1 penicilline, 100 µg ml-1 streptomycin, 2 mM L-glutamine (PAA) and NGF (50 ng/ml), plated on microscope cover glasses coated with laminin (20 µg/ml) and stored at 34°C in a humidified 5% CO_2_-atmosphere. Neurons were transferred into extracellular solution containing NaCl 137.6 mM, KCl 5.4 mM, MgCl_2_ 0.5 mM, CaCl_2_ 1.8 mM, glucose 5 mM and HEPES 10 mM (Roth, Karlsruhe, Germany), loaded with the fluorescent dye FURA−2 AM (1 µM; Biotrend, Köln, Germany). Fluorescence was measured using an inverted microscope (IX-81 with CellˆR, Olympus, Hamburg, Germany) and an ORCA-R2 CCD camera (Hamamatsu Corp., Bridgewater, NJ, USA). After alternating excitation with light of 340 nm and 380 nm wavelength, the ratio of the fluorescence emission intensities at 510 nm (340 nm/380 nm [510 nm]) was calculated and digitized at 0.5 Hertz. This fluorescence ratio is a relative measure of intracellular calcium concentration [33]. A cell was considered an excitable neuron and then evaluated only when significantly responding to application of capsaicin and/or depolarization by high potassium solution (140 mM). For analysis of absolute change in ratio, the baseline value was subtracted from the peak. Relative change was calculated by dividing the peak value by the preceding baseline. An increase by >23% was regarded as a significant response [32]. Each single slide investigated was regarded as an independent experiment where mean responses of the Capsaicin-sensitive and –insensitive subpopulations of neurons were determined. Nine independent experiments were performed on acutely dissociated DRG neurons obtained from two different male animals using NOD; eight from two other rats with dopamine.

**Figure 7:**
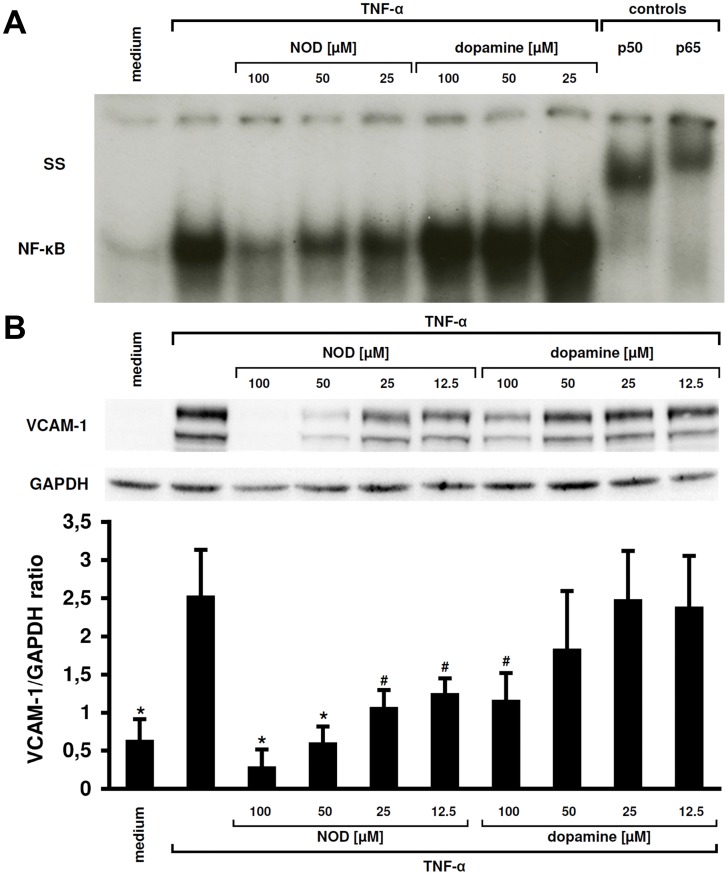
Influence of NOD on NF-κB activation and VCAM-1 expression in vitro. **A.** Influence of NOD and DA on TNF-α-mediated NFκB activation in proximal tubular epithelial cells (PTEC). While NOD dose-dependently inhibited NFκB activation this was not observed for DA when tested at equimolar concentrations (SS: supershift). **B.** Dose-dependent modulation of VCAM-1 expression by NOD. In line with the observed inhibition of NFκB, it was found that VCAM-1 expression on PTEC was more profoundly affected by NOD compared to DA. The upper panel shows a representative westernblot, in the lower graph 3 independent experiments were analysed by densitometry. *: P<0.01, #: P<0.05, all compared to TNF-α in the absence of NOD or DA.

### Electrophoretic Mobility Shift Assay (EMSA)

To assess the anti-inflammatory potential of DA and NOD, the ability of both compounds to inhibit NFκB was investigated in nuclear extracts from TNFα stimulated human proximal tubular epithelial cells (PTECs), but also nuclear extracts prepared from renal tissue. Isolation and cell culture of PTECs was performed as described [34]. PTECs were stimulated for 24 hrs with TNFα in the presence or absence of various DA or NOD concentrations (0–100 µM). Renal tissue obtained 1 day after induction of AKI was homogenized (4 randomized kidney samples of each group). Protein concentrations were determined by Bradford assay. EMSA was performed as previously described [35]. In each experiment specificity of binding was demonstrated by adding cold consensus or mutated NF-κB oligonucluotides to the nuclear extracts. In addition, supershifts were performed by adding anti-p65 and p55 antibodies to the samples. DNA-protein complexes were separated on 5% non-denaturating polyacrylamide gels electrophoresed in low ionic strength buffer and visualized by autoradiography.

**Figure 8:**
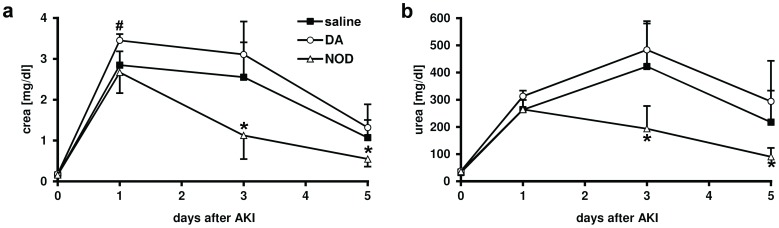
Renal function after induction of acute renal failure. **A.** Serum creatinine revealed a significantly improved renal function in the NOD group compared to the dopamine treated group (DA) at day 1 (^#^NOD vs. DA; P<0.01). Creatinine levels at day 3 and 5 after induction of AKI were significantly lower in NOD than both dopamine and saline control group (*NOD vs. DA/NaCl;P<0.001). **B.** Similar results could be observed by serum urea measurements. NOD showed significantly lower levels on day 3 and 5 (*NOD vs. DA/NaCl; P<0.001).

### Western Blotting of Epithelial Cells

PTECs were harvested and protein concentrations were measured using Coomassie-Reagent (Pierce, Rockford, USA). Samples (20 µg protein extract) were processed for blotting following standard procedures [36]. Thereafter, the blots were incubated with polyclonal anti-VCAM-1 (R&D Systems, Wiesbaden, Germany). In a second step membranes were incubated with the appropriate HRP conjugated secondary antibody (Jackson ImmunoResearch, MD, USA). Proteins were visualized using chemoluminescence technology according to the manufacturer’s instructions (Pierce, Rockford, IL). To confirm equal protein loading, membranes were re-probed with monoclonal anti-GAPDH antibody (Abcam, Cambridge, UK).

**Figure 9:**
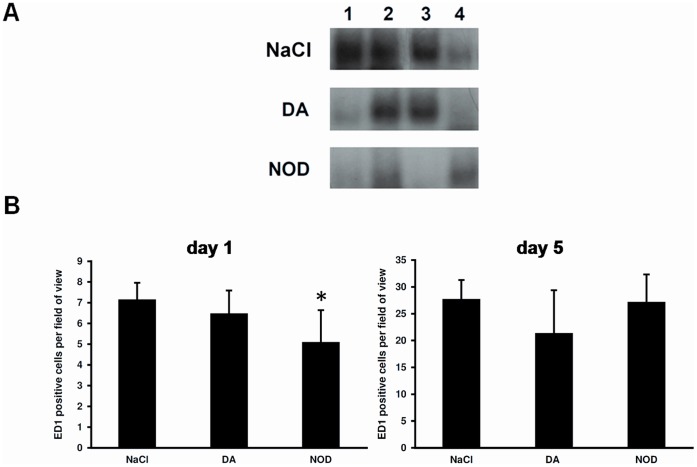
Influence of NOD on the activation of NFkB in vivo and infiltration of immune cells after ARF. **A.** One day after AKI NFκB in renal tissue was strongly activated in 3 out of 4 NaCl treated rats. In both NOD or DA treated animals 2 out of 4 displayed NFκB activation. No statistical analysis was performed. **B.** After one day, renal inflammation revealed that NOD significantly reduced the amount of ED1 positive cells compared to saline control (NaCl vs. NOD: *P<0.01, left panel). No significant differences between the groups at day 5 could be observed with ED1 positive cells (right panel).

The detection of TRPV1 in lysates of PTECs was performed using a rabbit anti-TRPV1 polycolonal primary antibody (Alomone Labs, Jerusalem, Israel) and the appropriate AP-conjugated secondary (Sigma, Germany). Proteins were visualized using NBT-BCIP substrate (Carl Roth, Karlsruhe, Germany). HEK cells transiently transfected with TRPV1 cDNA construct were used as positive control to indicate molecular weight of TRPV1.

**Figure 10:**
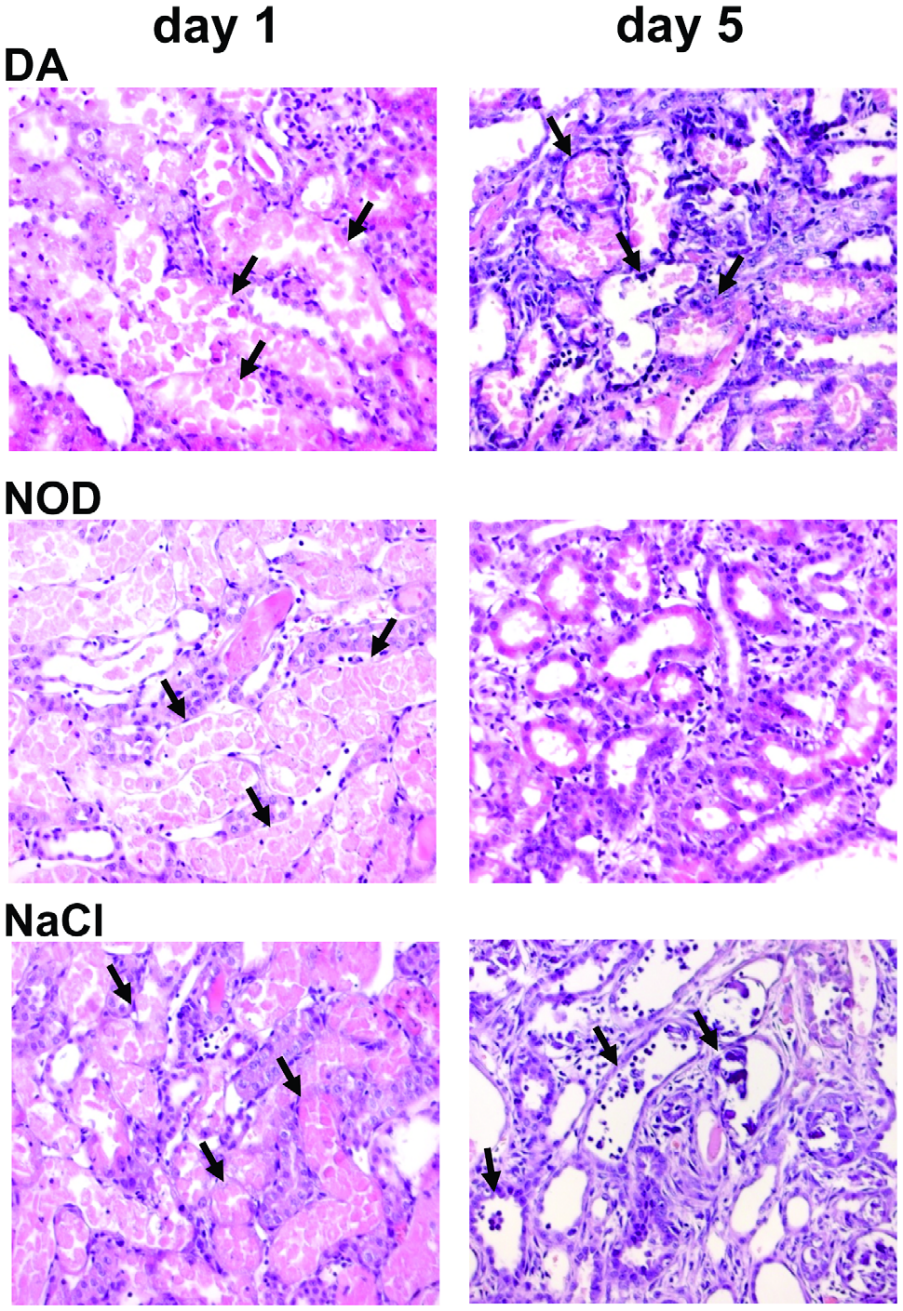
Histology (H&E stain) of proximal tubule segment S3 (pars recta) on day 1 and day 5. On day 1 there are no significant differences between the groups (left H&E stains). Cellular swelling, tubular epithelial necrosis and apoptosis are detectable at segment S3 at the pars recta of proximal tubules in all groups (see arrows). At day 5 (right H&E stains) in NaCl and DA groups revealed cellular debris in the tubular lumen, apoptosis, necrosis, shedding of the proximal tubule brush border with intraluminal calcifications and desquamation of cells (see arrows). In contrast in the NOD group proximal tubules show marginal calcifications and less destruction of epithelial cell lining with marked repopulation of tubular epithelial cells.

### Acute Kidney Injury

Animals were anaesthetised with xylazine (Rompun 2%®, Bayer Vital GmbH, Leverkusen, Germany) and ketamine (Ketamin 10%®, Intervet GmbH, Unterschleißheim, Germany), heparinized (100 IE, Heparin-Natrium ratiopharm®, Ratiopharm GmbH, Ulm, Germany) and subsequently injected intravenously via a tail vein with DA (Dopamin Fresenius (50 mg/5 ml), Fresenius Kabi GmbH, Bad Homburg, Germany), equimolar concentrations of NOD (Novaliq GmbH, Heidelberg, Germany) or NaCl (NaCl 0.9%, Fresenius Kabi GmbH, Bad Homburg, Germany), one hour before the renal artery was clamped for 45 min. The contra-lateral kidney was nephrectomised. Immediately before releasing the arterial clamp the animals received a second bolus i.v. injection of DA or NOD. The cumulative dose for DA and NOD was 360 µg and 670 µg respectively. Total intravenously applied volume was 600 µl in all animals. Adequate reperfusion after clamp opening was evaluated macroscopically. Renal function was assessed in all animals by serum creatinine and serum urea measurements on day 0, 1, 3 and 5 with an enzymatic method using a Hitachi 917 Autoanalyzer (Roche, Mannheim, Germany). Kidneys were harvested 1 or 5 days after induction of AKI for histology and to assess renal inflammation.

### Quantitative PCR

Snap-frozen renal tissue samples harvested 1 or 5 days after induction of AKI were homogenized. A total of 1 µg of RNA was reverse transcribed into cDNA. Quantitative PCR was performed on a real-time PCR platform (AB 7900HT) using TaqMan® probes for ICAM-1(Rn00564227_m1), VCAM-1 (Rn00563627_m1), E-selectin (Rn00594072_m1) (Applied Biosystems, Germany). Amplification was performed as proposed by the manufacturer. All samples were normalized for an equal expression of GAPDH.

### Histology and Immunohistochemistry

Kidneys were investigated 1 and 5 days after induction of AKI. Paraffin embedding of renal tissue, also hematoxylin and eosin (H&E) staining was performed using routine procedures. H&E stains were analyzed by a blinded pathologist. For immunhistochemical analysis the sections were incubated with ED-1 monoclonal antibody for detection of monocytes and macrophages (Linaris, Wertheim, Germany). Sections incubated with murine or rabbit IgG were used as negative controls. Standard avidin-biotin complex staining was performed according to the manufacturer’s instructions (Vector, Burlingame, CA, USA). ED-1 positive cells were counted by two blinded investigators, 20 microscopic fields per kidney were evaluated.

TRPV1 in frozen kidney sections was detected using rabbit anti-TRPV1 polyclonal primary antibody (Alomone) and secondary anti-rabbit Cy3 (Jackson ImmunoResearch, PA, USA). For double immunofluorescence labelling of TRPV1 and the neuronal marker protein gene product 9.5 (PGP 9.5), the sections of the kidney were fixed with 4% paraformaldehyde, blocked with 10% FCS in PBS and first incubated with guinea-pig anti-TRPV1 (Neuromics, Edina, MN, USA), which was detected with anti-guinea-pig-FITC (Jackson ImmunoResearch). The sections were then incubated with rabbit anti-PGP9.5 (Novus, Littleton, CO, USA) which was detected with anti-rabbit-Cy3 (Jackson). PTECs were stained with the guinea-pig anti-TRPV1 and visualized with secondary anti-guinea-pig-Cy3 (Jackson). Omission of primary antibody was used as negative control to exclude non-specific binding of the secondary. Photographs were taken using the IX-81 (Olympus) or a TCS SP5 DS laserscanning microscope (Leica Microsystems, Mannheim, Germany).

### Statistical Analysis

Data are expressed as mean ± standard deviation. For renal function, PCR-analysis and immunohistochemistry statistical analysis was performed using the Kruskal-Wallis test with option for multiple comparisons, calcium imaging data were analysed using Student’s t-test. For histology (H&E) Fisher’s exact test was used. Statistical analysis of westernblot experiments was performed by densitometry of at least 3 different experiment and by applying a Student’s t-test. A *P-* value of less than 0.05 was considered as significant.

## Results

### NOD but not DA Excites Peripheral Nerves Via Activating TRPV1

Immunofluorescent staining of renal tissue was clearly positive for some but not all tubular structures (Fig. 2A, arrow). In addition, there was a faint punctuated expression in the mesangial area of glomeruli noted (Fig. 2 upper panel, arrow head). At higher magnification PGP9.5 expressing peripheral nerve fibers localized between renal tubuli and some of the tubuli showed a positive staining for the capsaicin receptor TRPV1 (Fig. 2B, middle panels).

Peripheral nerve fibers originate from the soma of dorsal root ganglion neurons (DRG), a tissue known to extensively express TRPV1 [37], [38]. Since the majority of kidney projecting DRG neurons do express functional TRPV1 [39] we tested whether NOD may also excite these capsaicin-sensitive primary sensory nerve cells by using the calcium imaging technique and the fluorescent dye FURA-2-AM [33]. In a total of nine experiments we examined 499 neurons of which 345 responded to 10 µM of capsaicin and 177 to 30 µM of NOD, as reflected by an increase in free intracellular calcium (see Fig. 3 for representative examples). The mean change in intracellular calcium - as measured by fluorescent ratio - in NOD sensitive neurons was an increase to 240±6%. Almost all NOD sensitive cells also responded to capsaicin (174 of 177). When higher concentrations of NOD (100 µM) were used 51±5% of the DRG responded to both NOD and capsaicin (mean ± SEM), while 33±4% responded to neither. The remaining 13±3% responded to capsaicin only; the proportion of NOD-sensitive, capsaicin-insensitive cells was negligible (3±2%). Hence the vast majority of capsaicin-sensitive neurons were also excited by NOD.

The competitive TRPV1-antagonist capsazepine (CPZ, 10 µM) [32], [37], [40] was used to verify TRPV1 specificity in NOD responses. Similar as described for other TRPV1 agonists [37], [40], repetitive application of 30 µM NOD caused marked tachyphylaxis demonstrated by a significant decrease in mean response (to 63±3% at the 2^nd^ and to 64±2% at the 3^rd^ stimulus, p<0.01, 2^nd^ or 3^rd^ versus 1^st^) (Fig. 4). Application of CPZ before and during the second NOD bolus completely abolished the increase in free intracellular calcium (Fig. 4, black trace and bars; n = 5) which did no longer reach the threshold for a specific calcium transient (dotted line in Fig. 4B). In contrast, DA did not increase intracellular calcium, neither in capsaicin-sensitive (Fig. 5; black bars; n = 413) nor in insensitive neurons (open bars; n = 182; eight experiments), even when concentrations as high as 1 mM of DA were used.

### Anti-inflammatory Effects are More Pronounced with NOD than with DA

Because we observed positive staining for TRPV1 on distinct tubuli in rat renal tissue, we assessed if cultured human renal proximal tubular cells (PTEC) express TRPV1. Both in immunofluorescence and in Western blotting TRPV1 expression was demonstrated (Fig. 6). We next compared the anti-inflammatory potential of NOD with that of DA by studying the propensity of both compounds to inhibit TNFα mediated inflammatory responses in PTEC. While NOD dose-dependently inhibited NFκB activation, inhibition was marginal when equimolar concentrations of DA were used. Maximal inhibition was achieved at 100 µM of NOD (Fig. 7 A). No inhibition of VCAM-1 expression was observed when PTEC were stimulated with TNFα in the presence of capsaicin (data not shown). Down-regulation of VCAM-1 protein by NOD was clearly more profound compared to DA (Fig. 7 B).

### NOD Ameliorates Acute Kidney Injury

Based on the above findings that NOD activates TRPV1 and has superior anti-inflammatory properties than DA, we next investigated if NOD or DA treatment improves renal function after ischemia induced AKI. To this end, a bolus injection with DA or NOD was applied one hour before clamping of the renal artery and after 45 minutes of warm ischemia prior to clamp release. As assessed by s-creatinine measurement, NOD did not significantly improve renal function 1 day after the onset of AKI (NOD vs NaCl, P>0.05), but renal function was significantly improved on day 3 and day 5 (NOD vs. DA/NaCl; P<0.01; Fig. **8** **A**). In contrast, treatment with equimolar concentrations of DA compared to NaCl showed a trend towards a worse renal function, but this did not reach statistical significance. Analogous findings for s-urea concentrations were observed on days 3 and 5 (NOD vs. DA/NaCl; P<0.001; Fig. **8** **B**).

One day after induction of AKI NFκB was strongly activated in 3 of 4 saline treated controls, while in 1 animal this was not observed. Although it seemed that NOD also blunted NFκB activation in all analyzed animals (n = 4), its effect in vivo was clearly less pronounced compared to the in vitro findings. Similarly, the differences between NOD and DA in this regard were not clear in vivo, as in both the NOD and DA treated animals 2 out of 4 displayed NFκB activation, albeit that in the former animals NFκB activation might be less strong. Because of the small sample size and the heterogenous effect of NFκB activation in the control saline group, statistical analysis was not performed (Fig. **9** **A**). In line with the possible small effect on NFκB activation in vivo, it was found that neither in NOD or DA treated rats, the expression of the adhesion molecules ICAM-1, VCAM-1 and E-selectin was affected, as demonstrated by qPCR analysis (data not shown). Similarly, neither DA nor NOD inhibited mRNA expression of inflammatory cytokines at any time of investigation (data not shown).

H&E stainings one day after AKI revealed necrosis and apoptosis in segment 3 of proximal tubuli (pars recta) in all groups, most likely as a consequence of the ischemic insult. However, 5 days after the onset of AKI in kidneys of NOD treated rats no necrosis or apoptosis was observed. This was in sharp contrast to DA or saline treated rats (apoptosis: saline: 7/7; DA: 5/7; NOD 0/7 (NOD vs. NaCl, P<0.01; NOD vs. DA, P<0.05); necrosis: saline: 7/7; DA: 4/7; NOD 0/7 (NOD vs. NaCl, P<0.01, NOD vs. DA not significant)). Also intraluminal calcifications were strongly diminished in NOD treated rats (NaCl: 7/7; DA: 6/7; NOD 2/7 (NOD vs. NaCl, P<0.05, NOD vs. DA not significant)) 5 days after AKI induction (Fig. **10** **).** It seems that a bolus application of NOD contributes to repair of damaged epithelium thereby restoring tubular epithelial integrity.

Immune histology performed one and five days after the onset of AKI revealed a significant reduction of infiltrated ED-1 positive monocytes/macrophages at day 1 in the NOD treated rats, but this was no longer significant at day five (Fig. 9 B).

## Discussion

In the present study we compared the efficacy of NOD and DA with respect to their ability to activate TRPV1, their ability to inhibit TNFα mediated inflammation and their ability to mitigate AKI. The main findings of this study are the following. Firstly, in vitro studies revealed that NOD, but not DA, dose-dependently activates TRPV1 receptors. In renal tissue TRPV1 positive nerve fibers and fiber networks showed a peritubular and vascular localization. In addition, TPRV1 was expressed on distinct tubuli in rat renal tissue and on cultured human proximal tubular epithelial cells (PTEC). NOD strongly impaired NFκB activation in TNF-α stimulated cultured PTEC resulting in a decreased expression of VCAM-1. This was not observed when PTEC were stimulated with TNF-α in the presence of capsaicin. In vivo inhibition of NFκB by NOD was not strong, and was not paralleled by a decrease in the expression of NFκB regulated genes in renal tissue obtained from rats suffering from ischemia induced AKI. Monocyte infiltration one day after the onset of AKI was reduced in NOD treated, but not in DA treated rats, but was not significantly different at day five. No significant difference in the expression of adhesion molecules and cytokines was observed at any time-point of investigation. Thirdly, AKI was mitigated in NOD -, but not in DA treated rats. This was reflected by a better tubular epithelial integrity and improved renal function.

There is ample evidence from controlled clinical data that the routine use of dopamine in the critically ill patients with impending or overt renal failure is no longer warranted [13], [14]. In agreement with those findings, our study also indicates that DA is not improving renal function in the setting of AKI in the rat. Nonetheless, we have made three important observations in this study: firstly NOD activates TRPV1, secondly NOD positively affects regeneration of tubular epithelium after I/R injury and thirdly NOD has superior intrinsic anti-inflammatory properties compared to DA.

The endogenous N-acyl-dopamine derivatives (NADD), i.e. N-oleoyl-dopamine or N-arachidonoyl-dopamine, can act as TRPV1 agonists [24], [41]. In the present study we now also show that the synthetic N-octanoyl-dopamine (NOD) behaves in a similar fashion, suggesting that TRPV1 activation might be a functional property of the whole class of N-acyl-dopamine derivatives. In line with previous findings [42], [43], we show that TRPV1 positive nerve fibers are found in the renal cortex. The majority of DRG neurons innervating the kidney are activated by capsaicin indicating that functional TRPV1 channels are expressed in those nerves [39]. Microvascular dysfunction related to the imbalance of vasoconstrictors and vasodilators in renal I/R injury leads to a reduction of microcirculatory oxygen supply with local inflammation and is believed to play a pivotal role in the pathogenesis of AKI [44]. Activation of TRPV1 induces the release of neuropeptides including calcitonine gene-related peptide (CGRP) and substance P (SP). These neuropeptides are known to be potent vasodilators in most vascular beds [45] and this in turn might contribute to attenuation of I/R injury [24], [46]. It should however be underscored that this study neither addresses as to whether the renoprotective effect of NOD is related to TRPV1 activation nor, if so, whether this is mediated via improved microvascular perfusion of the kidney. Further studies with the use of TRPV1 knock-out animals are therefore still warranted.

The role of TRPV1 in modulation of inflammatory processes is controversially discussed. While Szabó et al [47] and Keeble et al [48] have shown that TRPV1 activation aggravates inflammation in arthritis models, the use of TRPV1 agonists in models of AKI, cardiac ischemia and sepsis was associated with a diminished inflammatory response [23], [24], [49]. The paradoxical role of TRPV1 in inflammation may be explained by desensitization and suppression of the channel by long lasting or repeated activation – a well known phenomenon used e.g. for the treatment of pain with TRPV1 agonists [50], [51]. The marked tachyphylaxis seen in DRG neurons during repeated application of NOD suggests that not necessarily activation of TRPV1 but desensitizing the TRPV1-dependent vasculature within the kidney may account for the renoprotection seen in our study. It should however be emphasized that by virtue of their ability to inhibit signalling pathways involved in inflammation, e.g. NFκB, NFAT and activator protein 1 signalling [27], [52], a number of TRPV1 agonists, including NADD, have potential anti-inflammatory properties independent of TRPV1. Apart from TRPV1 mediated organ perfusion, the beneficial effect of NADD on I/R injury [23] might therefore also involve TRPV1 independent mechanisms.

There is ample evidence for the involvement of NFκB in renal pathology after ischemia induced AKI, as the use of NFκB decoys proved to be beneficial in both AKI [53] and transplantation models [54]. Indeed NFκB was far better inhibited in vitro by NOD than by DA in cultured PTEC. This was paralleled by the finding that VCAM-1 expression was more profoundly down-regulated by the former when PTEC were stimulated with TNFα. PTEC express TRPV1, but the inhibition of NFκB by NOD was unlikely mediated via TRPV1 as capsaicin was not able to prevent VCAM-1 expression. Unexpectedly, in vivo the anti-inflammatory properties of NOD were modest. With the exception of a possible inhibitory effect on NFκB in some but not all animals and the reduction in monocyte infiltration at day 1, the expression of none of the tested inflammatory mediators, e.g. adhesion molecules and cytokines, was reduced in NOD treated rats.

Both DA and NOD are able to act as reducing agents and therefore have the intrinsic ability to scavenge reactive oxygen species. We have previously shown in a RCT that donor pretreatment with DA preserves early graft function after kidney transplantation particularly in kidney grafts that were exposed to prolonged cold ischemic time (16). An adequate anti-oxidant defence may be of major importance in a number of renal pathologies including ischemia induced AKI [55], [56], [57], [58]. Redox activity of DA and NOD depends on the ability to oxidize the catechol structure, and on the metal chelating properties hereof [59]. However, protection from oxidative stress relies on the molecule’s ability to diffuse into cells [60]. DA and NOD markedly differ in their hydrophobic nature, which explains why cellular up-take of NOD is more efficient [31]. While DA is rapidly metabolized by monoamine oxidase (MAO) and catechol-O-methyltransferase (COMT), it is not yet known if these enzymes are able to degrade NADD. It remains to be seen if the structural modifications of the DA molecule which enhance its relative hydrophobicity and thereby determine its pharmacokinetic and –dynamic properties can explain or contribute to the renoprotective effect of NOD in AKI.

### Conclusions

In conclusion, we have shown in an experimental model that NOD mitigates AKI and improves renal function. Therefore, our findings have a high clinical relevance since at present no adequate treatment for AKI exists for critically ill patients. Although our data and that from other pre-clinical studies [23] show promising results on the use of NADD and other vanilloid receptor related agonists in AKI, further studies are warranted to address the underlying renoprotective mechanism including the role of the capsaicin receptor TRPV1.
