# Correction: N-octanoyl-Dopamine Is an Agonist at the Capsaicin Receptor TRPV1 and Mitigates Is Chemia-Induced Acute Kidney Injury in Rat

**DOI:** 10.1371/annotation/07b07d73-c132-46a6-b267-b1694d20ae36

**Published:** 2012-08-23

**Authors:** Charalambos Tsagogiorgas, Johannes Wedel, Maximilia Hottenrott, Michael O. Schneider, Uta Binzen, Wolfgang Greffrath, Rolf-Detlef Treede, Bastian Theisinger, Sonja Theisinger, Rüdiger Waldherr, Bernhard K. Krämer, Manfred Thiel, Peter Schnuelle, Benito A. Yard, Simone Hoeger

There was a typographical error in the title and citation. The correct title is, "N-octanoyl-Dopamine Is an Agonist at the Capsaicin Receptor TRPV1 and Mitigates Ischemia-Induced Acute Kidney Injury In Rat." The correct citation is:

Tsagogiorgas C, Wedel J, Hottenrott M, Schneider MO, Binzen U, et al. (2012) N-octanoyl-Dopamine Is an Agonist at the Capsaicin Receptor TRPV1 and Mitigates Ischemia-Induced Acute Kidney Injury in Rat. PLoS ONE 7(8): e43525. doi:10.1371/journal.pone.0043525

